# 

**Published:** 1907-03

**Authors:** 


					CONTiENTS.
Page
Prognosis.'
--Smith, M.D., F.R.C.P., F.R.S.
Cerebrospinal Fever (Epidemic Cerebro-Spinal Meningitis).
By
D. S. Davies, M.D.Lond., and I. Walker Hall, M.D.Vict.
14
Operations for Deflections and Spurs of the Nasal Septum, with
special reference to Sub-mucous Resection.
By Patrick
Watson Williams, M.D. Lond.
Two Cases of Ruptured Intestine.
By Harold I*. Mole, h K.U.s.
38
Concluding Notes on a Case of Splenomegalic Cirrhosis in a
Child aged seven years.
By Edward Cecil Williams, M.ii. Lantab.
With post-mortem notes by J. M. Fortescue-Brickdale, M.A.,
M.D. Oxon.
43
Acquired Angioma of the Liver.
By A. Lewin Sheppard, M..b.,
B.S. Durh.
46
The Sanatorium Treatment of Pulmonary Tuberculosis?Is it a
Success?
By D. J. Chovvry Muthu, M.D.
5?
oome Notes on Stvracol.
By Charles Reinhardt, M.D.
54
Progress of the Medical Sciences :
Medicine.
J. R. Charles, M.A., M.D., B.C. Cantab.
57
ourgery.
E. W. Hey Groves, M.B., ts.sc. Lona.,
bi
r'atholoey.
I. Walker Hall, M.D. Vict.
67
reviews of Books
og
| tentorial Notes
76
Notes on Preparations for the Sick
83
J he Library of the Bristol Medico-Chirurgical Society   85
?yieetiners of Societies
?7
Obituary Notices
go
Local Medical Notes
yj

				

